# Characterisation of novel protein families secreted by muscle stage larvae of *Trichinella spiralis*^☆^

**DOI:** 10.1016/j.ijpara.2008.09.012

**Published:** 2009-04

**Authors:** David B. Guiliano, Yelena Oksov, Sara Lustigman, Kleoniki Gounaris, Murray E. Selkirk

**Affiliations:** a Division of Cell and Molecular Biology, Department of Life Sciences, Imperial College London, London SW7 2AY, UK; b Laboratory of Electron Microscopy, Lindsey F. Kimball Research Institute, New York Blood Center, New York, NY 10021, USA; c Laboratory of Molecular Parasitology, Lindsey F. Kimball Research Institute, New York Blood Center, New York, NY 10021, USA

**Keywords:** *Trichinella spiralis*, Nurse cell, Secreted proteins, Immunohistochemistry, Expressed sequence tags, Zinc-dependent metalloprotease

## Abstract

Proteins secreted by *Trichinella spiralis* have a potential role in remodelling host skeletal muscle. However, whilst many parasite-secreted proteins have been identified, it has rarely been demonstrated that these are secreted into the nurse cell. Using an informatics-based analysis, we have searched the *T. spiralis* expressed sequence tag (EST) datasets for cDNAs encoding potential secreted proteins. Here we describe the characterisation of three of the top candidates isolated from our analysis, termed secreted from muscle stage larvae (SML)-1, -2 and -3. All three proteins were demonstrated to be secreted by muscle stage larvae, and immunohistochemical analysis established that SML-1 and -2 are secreted into developing nurse cells. We also show that SML-2 is processed from a precursor into smaller peptides by a metalloprotease contained within *T. spiralis*-secreted products. With the identification of these and other secreted proteins, we now have molecules to test in functional assays designed to dissect molecular features of the developing nurse cell.

## 1 Introduction

Nematode parasites of the genus *Trichinella* infect a wide variety of mammalian hosts, including humans. During its life cycle the parasite colonises two primary tissue sites, the intestinal epithelium (adults) and the skeletal muscle (muscle stage larvae, mL1s). Trichinellids are unique because in both of these environments, the nematodes occupy intracellular niches. Newborn larvae (NBL) released by adults in the intestine rapidly exit the lumen and migrate via the circulatory system until they invade a skeletal muscle fibre. Once established in the myofibre cytoplasm, NBLs initiate a remodelling process which, over the course of a few weeks, transforms it into a new structure termed the ‘nurse cell’ (Despommier, 1993). During this transformation process, the myofibre undergoes a dramatic developmental shift that radically alters its transcriptome, proteome and cellular architecture. Myofibre nuclei re-enter cell cycle, replicate their DNA but remain blocked before mitosis in G2/M (Jasmer, 1993). Muscle protein expression is repressed and novel transcripts such as syndecan-1 are induced (Jasmer, 1993; Beiting et al., 2006). Angiogenic factors such as vascular endothelial growth factor (VEGF) are produced by parasitised fibres, new blood vessels are recruited forming a circulatory rete (Capo et al., 1998) and large numbers of mononuclear cells invade the adjacent tissue around the fibre (Beiting et al., 2004). Finally, in encapsulating species such as *Trichinella spiralis*, collagens (types IV and VI) and other extracellular matrix proteins are synthesised and form a capsule around the nurse cell (Polvere et al., 1997).

The nurse cell and its resident nematode(s) form a stable complex, which can survive for long periods in immune-competent hosts (Despommier, 1993). The nurse cell phenotype has been studied using a number of methodologies, yet the molecular basis of the transformation process remains largely uncharacterised. *Trichinella*-derived glycoproteins can be detected within the cytoplasm of the myofibre a few days after invasion. These secreted proteins (SPs) are synthesised in a specialised secretory organ called the stichosome that begins to develop in the larvae a few days after invasion of the muscle fibre. Irradiation of NBLs prior to infection causes a number of developmental defects, including inhibition of stichosome genesis and reduced secretion of some parasite products into the nurse cell (Jasmer and Neary, 1994; Jasmer et al., 1994). Despite these defects the transformation of the invaded muscle fibre still occurs, although there is a distinct delay in the onset of early features such as cell cycle re-entry (Jasmer and Neary, 1994). This suggests that while larval development and secretion of parasite products into the muscle fibre may be linked to the early stages of nurse cell transformation, the factors synthesised within the stichosome are not required for this process to occur. Similarly, manipulation of parasite protein secretion, through post-infection treatment of animals with mebendazole, supports a role for SPs in nurse cell maintenance (Yao et al., 1998). Treatment of nurse cells with this drug result in depletion of parasite-derived SPs which coincides with alterations in hypertrophic nuclei and changes in nurse cell markers such as reduced acid phosphatase levels. Thus, while the role parasite SPs play in the nurse cell genesis remains controversial, there is some evidence supporting their involvement in the transformation process and maintenance of homeostasis within the mature nurse cell.

A number of directed studies and proteomic analyses have identified a plethora of enzymatic activities and at least a dozen components of *Trichinella* mL1s SP (Zarlenga and Gamble, 1990; Gounaris et al., 2001, 2004; Kuratli et al., 2001; Tan et al., 2001; Selkirk et al., 2004; Robinson and Connolly, 2005; Bruce et al., 2006; Bruce and Gounaris, 2006). However, with the exception of the 43 kDa glycoprotein (gp43) and other antigenically related proteins, there is little direct evidence that these proteins are secreted into the nurse cell (Despommier et al., 1990; Vassilatis et al., 1992; Yao et al., 1998). This has hampered the development of functional assays to dissect the nurse cell phenotype, thus limiting the scope for expanding our understanding of this fascinating biological system.

To begin to address this issue, we have undertaken an informatics and transcriptomics-based analysis to identify novel *T. spiralis*-secreted proteins, which could potentially interact with host skeletal muscle. Here we describe the identification and initial characterisation of three candidate molecules which we have named secreted from muscle stage larvae (*sml*) 1, 2 and 3. We further show that two of these proteins SML-1 and -2 are secreted by the parasite into the nurse cell.

## 2 Materials and methods

### 2.1 Parasites

Mice and rats were purchased from Harlan (Oxford, UK). Food and water were available *ad libitum*. All procedures involving and care and maintenance of animals were approved by the Imperial College Ethical Review Committee and performed under licence from the UK Home Office. Muscle stage larvae of *T. spiralis* (T1 ISS930) were recovered from Sprague–Dawley rats 2 months after oral infection, cultured in serum-free RPMI-1640 for 72–80 h and SP collected as previously described (Gounaris et al., 2001). Adult worms were collected from infected rat intestines 2 or 6 days p.i. by sedimentation in a Baermann funnel. Day 6 adults were cultured for 3 days in serum-free RPMI-1640 and SP collected as described previously (Gounaris et al., 2001). NBL were separated from adult worms by sedimentation and immediately frozen or fixed for subsequent analysis.

For inhibition of post-translational processing of SML-2, 150,000 mL1s were cultured for 72 h in RPMI supplemented with protease inhibitors as described in the legend to Fig. 3. Because of their instability, additional PMSF (phenylmethylsulphonyl fluoride), TLCK (*N* α-Tosyl-Lys-chloromethylketone) and NEM (*N*-ethylmaleimide) were supplemented at day 2 of culture. Supernatants were immediately frozen and concentrated using a microcon YM-3 (Millipore). The viability of mL1s cultured in protease inhibitors was monitored daily.

Soluble whole worm extracts (SXT) were prepared by disruption of mL1s or adults in a custom-made Bessman tissue pulveriser and protein extraction buffer (25 mM Hepes, pH 7.5, 1.5% *n*-octyl β-d-glucopyranoside and protease inhibitors). Parasite protein extracts were incubated on ice for 30 min, briefly sonicated, centrifuged at 15,000*g* for 15 min and supernatants stored at −20 °C. The protein content of concentrated SP and SXTs was determined by the BCA (Pierce) or Bradford (BioRad) microplate assay.

### 2.2 Analysis of *T. spiralis* expressed sequence tag (EST) datasets and identification of SMLs 1, 2 and 3

*Trichinella spiralis* EST datasets (4272 predominately mL1s ESTs) were downloaded from GenBank and clustered locally using the CLOBB algorithm (Parkinson et al., 2002), followed by subsequent analysis of adult and NBL EST datasets (Mitreva et al., 2004). Two thousand and eighty-four consensus sequences generated from the EST clusters were compared to the non-redundant nucleic acid and protein sequences within GenBank and other nematode EST sequence datasets. Potential open reading frames (ORFs) were predicted from the consensus sequences using Phrap (Gordon et al., 2001; Parkinson et al., 2004), and these ORFs were subsequently manually curated as poor sequence quality at the 5′ regions of the ESTs frequently caused frame shifts which removed N-terminal sections of the proteins, including signal peptides. The ORFs were then searched against signal-P, PSORT, PROSITE, and SMART to obtain predicted sub-cellular localisation and protein domain architecture data (Nakai and Horton, 1999; Schultz et al., 2000; Bendtsen et al., 2004; Hulo et al., 2006). One hundred and four ORFs with predicted signal peptides were identified and then ranked using a combination of the frequency of representation in the EST dataset and the postulated sub-cellular localisation. Thirty-nine of the candidates are members of 91 clusters with five or more ESTs (42%).

### 2.3 Sequence confirmation and expression analysis of SMLs

For each candidate protein, the corresponding cDNA sequence was identified in GenBank: SML-1, ps38e09.y1; SML-2, ps31d05.y2 and ps94b11.y1; and SML-3, ps32b06.y1 (GenBank Accession Numbers BG353871, BG438572, BQ542163, BG353368). It appeared that the predicted ORF of each of the cDNA clones contained the full-length coding region of the genes. Nonetheless the cDNA clones were obtained from Irina Roko at Washington University (St. Lewis, USA) using the nematode EST clone provision service, and the sequences confirmed using vector and gene-specific primers (when necessary). The verified sequences were deposited in GenBank (SML-1: EU867515, SML-2: previously submitted under DQ777102, and SML-3: EU867516). The expression of SMLs 1, 2 and 3 in different *T. spiralis* life cycle stages was analysed by reverse transcriptase (RT)-PCR. RNA was isolated from *T. spiralis* by flash freezing nematodes in Trizol (Invitrogen) and disrupting them using a custom-made Bessman tissue pulveriser according to the manufacturer’s protocols. Isolated RNA was treated with RNase-free DNAse and then purified using RNAeasy mini column purification (Qiagen). Reverse transcription was performed using 2 μg of total RNA, Superscript II and oligo (dT) (Invitrogen) according to the manufacturer’s protocols. PCR was performed with gene-specific SML primers (for sequences see Supplementary Table S1), standard reaction conditions and PCR cycle parameters (one cycle 95 °C for 3 min; 35 cycles at 94 °C for 30 s, 55 °C for 30 s, 72 °C for 1 min; one cycle 72 °C for 10 min). PCRs were run on 2% agarose gels, visualised with ethidium bromide and documented. *Trichinella spiralis* alpha tubulin was used as an internal positive control and no RT reactions were used as negative controls (GenBank sequence EU867518; primer sequences in Supplementary Table S1).

### 2.4 Bacterial expression of SMLs and production of murine polyclonal antiserum

ORFs corresponding to predicted mature peptides of SMLs 1 (amino acids 36–279), 2 (amino acids 19–406) and 3 (amino acids 21–117) were fused to a poly-histidine epitope tag (His-tag) by cloning into the NdeI and XhoI sites of the *Escherichia coli* expression vector pET-29b (Novagen). The primers used for cloning are listed in Supplementary Table S1. Protein was expressed using either standard IPTG induction or via culture in Overnight Express media (Merck Chemicals). Insoluble recombinant proteins were purified under denaturing conditions using nickel affinity chromatography and His-Bind Quick 900 cartridges (Merck Chemicals). Denaturing agents were then removed by dialysis into PBS and protein solutions concentrated and quantified by the BCA or Bradford assay. SDS–PAGE and coomassie blue staining was used to assess recombinant protein purity.

Polyclonal antisera to SML-1, -2 and -3 were raised in BALB/c mice using a standard immunisation protocol and Freund’s incomplete adjuvant. Antibody responses to SMLs were screened by ELISA and Western blotting prior to collection of serum.

### 2.5 SDS–PAGE and Western blotting

Protein samples were resolved by 12% or 15% SDS–PAGE. For Western blot analysis, 10 μg of protein samples were resolved by SDS–PAGE, transferred onto polyvinylidene fluoride (PVDF) membranes, blocked with 5% skimmed milk powder/PBS/0.1% Tween-20 for 1 h prior to incubation at 4 °C overnight with anti-SML murine polyclonal antisera diluted (1:200) in blocking solution and pre-absorbed with *E. coli* lysate (Promega). Antibody binding was visualised using standard procedures with horseradish peroxidase (HRP)-conjugated anti-mouse IgG secondary antibody (BioRad) and chemiluminescent detection (ECL, Amersham).

Deglycosylation of 10 μg of SP using *N*-Glycosidase F (PNGase F, NEB) was performed according to the manufacturer’s protocol. Briefly, protein samples were denatured, 50 U ml^−1^ of enzyme added and the reaction incubated for 12 h at 37 °C. Following SDS–PAGE, products were visualised by Western blotting. For analysis of proteolytic processing of SML-2, 5 μg of protease inhibitor-treated mL1s SPs were resolved by SDS–PAGE and visualised by Western blotting.

### 2.6 Cellular localisation of native SMLs

Thigh muscle from rats infected with *T. spiralis* was collected 18, 24, or 28 days p.i. Tissue or isolated nematodes were either fixed in 10% neutral buffered formalin overnight and then embedded in paraffin according to standard protocols or embedded in O.C.T. (Optimal Cutting Temperature) compound and snap frozen in isopentane cooled in liquid nitrogen. Thin sections (8 μm) were cut from tissue blocks onto superfrost slides. Frozen sections were fixed in ice-cold methanol for 15 min, air dried and then stored at 4 °C. For immunofluorescence, methanol-fixed frozen sections were blocked in PBS/5% normal goat serum/5% BSA for 1 h at room temp and then incubated overnight at 4 °C with murine anti-SML antisera diluted in blocking solution. Sections were then washed thoroughly with wash buffer (PBS/0.1% Triton X-100), incubated with tetramethyl rhodamine iso-thiocyanate (TRITC) conjugated goat anti-mouse IgG for 1 h, washed thoroughly with wash buffer, mounted with 4′,6-diamidino-2-phenylindole (DAPI) counterstain and visualised using a Nikon Eclipse E400 fluorescence microscope.

Paraffin sections were de-waxed with Histoclear and rehydrated. Antigen retrieval was performed by microwaving sections in a solution of either citrate based or high pH antigen unmasking solutions (Vector Labs) for 15 min. Endogenous peroxidase activity was blocked by incubation in 2% H_2_O_2_. Sections were blocked in PBS/5% normal goat serum/5% BSA for 1 hr at room temperature and endogenous biotin and streptavidin binding sites blocked using a commercial blocking kit (Vector Labs). Sections were incubated overnight at 4 °C with murine anti-SML diluted in blocking solution, washed thoroughly with wash buffer, incubated with biotin-conjugated goat anti-mouse IgG for 1 h, washed again and detected with Streptavidin–HRP complex and DAB substrate (3,3′-diaminobenzidine, Vector Labs). Sections were counterstained with Mayer’s haematoxylin, mounted with DPX mounting media (BDH) and visualised.

For immuno-gold transmission electron microscopy (TEM), adults and NBL were fixed for 60 min in 0.25% glutaraldehyde, 1% sucrose in 0.1 M phosphate buffer pH 7.4, and then processed for immunoelectron microscopy as previously described (Lustigman et al., 1991, 1992). Muscle stage larvae were fixed in 4% paraformaldehyde overnight and then processed as above. For immuno-localisation of the native parasite antigen corresponding to SML-1, -2 and -3, thin sections (70 nm) of embedded worms were incubated with murine polyclonal antisera, followed by incubation with 15 nm gold particles coated with goat anti-mouse IgG (Amersham Life Sciences, Piscataway, NJ). Pre-immune serum was used as the control.

### 2.7 Analysis of SML gene families

The *T. spiralis* EST datasets and a local copy of the partially assembled *T. spiralis* genome (http://www.genome.wustl.edu/genome.cgi?GENOME=Trichinella%20spiralis) were searched with the confirmed SML sequences using the BLAST algorithm (Altschul et al., 1997). Searches of these datasets identified two additional members of the *sml-2* gene family and a number of potential *sml-3*-like sequences. Genomic contigs containing these sequences were then annotated in Artemis (Rutherford et al., 2000), and gene predictions were made based on homology to the confirmed *sml* sequences and ESTs in GenBank. The predicted cDNAs and the protein sequences they encode are listed in Supplementary Table S2 and were compared using ClustalX (Chenna et al., 2003). For the *sml-3*-like gene fragments, the sequence identifiers are derived from the contig numbers they are found within. In two contigs, multiple *sml-3*-like sequences were identified.

## 3 Results

### 3.1 Identification and expression analysis of SMLs 1, 2 and 3

The informatics analysis of the *T. spiralis* EST dataset identified dozens of potential candidate molecules to examine in our search for proteins secreted into the nurse cell. These candidates were then searched for other features such as protein localisation signatures (nuclear localisation motifs, trans-membrane domains, etc.) and ranked based on their frequency of representation in the EST datasets, with the most abundant transcripts receiving the highest rank scores. The top five ranked candidates are listed in Table 1. Candidates lacking clear homology to proteins of known function in GenBank were given the designation secreted by muscle stage larvae (SML).

The first and top candidate on the list was predicted to encode a 30-kDa protein containing granulin-like repeats. This transcript and its protein product, which we have termed GRN-1, will be discussed further in a subsequent publication, but the confirmed sequence from the EST clone ps26g11.y1 (BG520554) has been submitted to GenBank (EU867517).

**Fig. 1:**
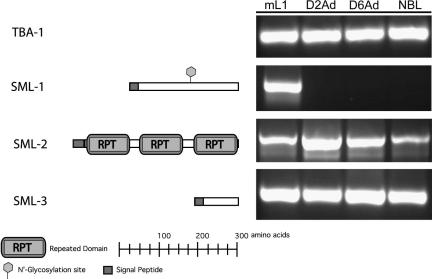
Bioinformatic and expression analysis of three *Trichinella spiralis* proteins secreted from muscle stage larvae (SMLs). This diagram shows features identified in predicted protein translations of SML-1, -2 and -3 whose transcripts were identified within the *T. spiralis* muscle stage larvae expressed sequence tag dataset. These include signal peptides, confirmed N-linked glycosylation sites, and a repeated protein domain. Reverse transcriptase (RT)-PCR showing the expression of these transcripts at different life cycle stages is also shown. mL1s: muscle stage larvae, D2Ad: day 2 adults D6Ad: day 6 adults, NBL: new born larvae. RT-PCR with *T. spiralis* alpha-tubulin (TBA-1) specific-primers was also performed to assess the quality of the cDNA used in the expression analysis.

The second ranked candidate on the list is a novel transcript predicted to encode a 31-kDa protein which we have designated *sml-1*. The mature peptide has no homologies to any other protein in GenBank but contains one potential glycosylation site (Fig. 1). Analysis with the protein localisation algorithm PSORT revealed that Reinhardt’s method for cytoplasmic/nuclear discrimination predicts that the mature SML-1 peptide will have a nuclear localisation (Nakai and Horton, 1999).

Candidate number three is the previously characterised 45 kDa glycoprotein with similarities to serine proteases (Arasu et al., 1994). Candidates number four (*sml-2*) and five (*sml-3*) were found to be identical to gene fragments previously submitted to GenBank by Despommier (9.10 GenBank Accession Number AAB48491.1 and 11.30 GenBank Accession Number AAB48488.1). *sml-2* is predicted to encode a 47-kDa protein with a tandem repeated domain and one potential glycosylation site (Fig. 1). *sml-3* encodes a novel 13 kDa protein, containing one potential glycosylation site but no homology to any other proteins in GenBank (Fig. 1).

Both SML-2 and SML-3 were recently identified in a proteomic analysis of mL1s secreted products (Robinson and Connolly, 2005). Robinson and colleagues further characterised SML-2, giving it the designation multi-domain ‘cystatin-like’ protein (MCD-1), after searches with InterProScan found each tandem repeated domain contained within the predicted ORF had low sequence homology to type II cystatins (Quevillon et al., 2005). Our own analysis of the SML-2/MCD-1 predicted ORF with BLAST and InterProScan searches failed to yield matches to cystatins or any other protein domains. However, comparison of SML-2/MCD-1 to the SMART and MEROPs databases indicate that the individual repeated domains have ∼25% similarity with rat cystatin E/M (Rawlings et al., 2008). As part of their analysis Robinson and colleagues also highlight several potential conserved active site residues within the each domain including the N-terminal glycine, degenerate Q-x-V-x-G and PW motifs, and four cysteine residues (Robinson et al., 2007). However, the lack of any functional data combined with the questionable sequence conservation makes the assignment of SML-2/MCD-1 to a group of cystatin-related proteins tenuous. To maintain consistent nomenclature within the literature we will adopt MCD as the designation for this protein family. A second SML-2/MCD-1 family member, whose predicted protein contains a potential secretory leader and two ‘cystatin-like’ domains, has recently been deposited in GenBank (Accession Number ABY59464, and NEMBASE cluster TSC01027). We have given this sequence the designation *mcd-3*.

Two protein sequences with 82% sequence identity to SML-3 isolated during a subtractive hybridisation screen were also recently submitted to GenBank (Liu et al., 2007). Analysis of SML-1, 2/MCD-1, and 3 transcript expression by RT-PCR revealed that SML-2 and -3 are expressed throughout the life cycle of the parasite, while SML-1 is specifically expressed in mL1s (Fig. 1).

### 3.2 Secretion and post-translational modification

**Fig. 2:**
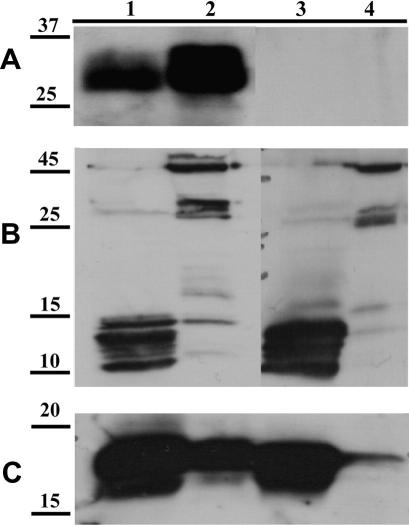
Demonstration of secretion of secreted from muscle stage larvae (SMLs)-1, -2, and -3 by *Trichinella spiralis*. Western blots of *T. spiralis* secreted proteins (SP) and soluble extracts (SXT) probed with murine polyclonal antisera to SML-1, -2 and -3 demonstrate they are secreted by parasites. (A) Western blot probed with anti-SML-1. (B) Western blot probed with anti-SML-2. (C) Western blot probed with anti-SML-3. 1: mL1s SP, 2: mL1s SXT, 3: AdD6 SP, 4: AdD6 SXT. Molecular mass markers are shown in kilodaltons. See Fig. 1 for abbreviations.

Recombinant SML-1, -2 and -3 were expressed as His-tag fusion proteins in *E. coli* and purified using nickel affinity chromatography. Recombinant proteins were then used to generate murine polyclonal antisera, which were used as probes in Western blots with parasite material. SML-1 was identified in both mL1s SPs and SXTs, with the native protein migrating at approximately 30 kDa (Fig. 2A). No SML-1 was detected in either extracts or secreted proteins of day 6 adults. Native SML-2 protein was identified in both extracts and SPs of mL1s and adults, although the reactive bands varied in these preparations (Fig. 2B). In extracts we detected a single band at 47 kDa and a doublet at 30 and 32 kDa. This contrasted with SPs, in which minimal binding to the 47 and 30/32 kDa species was observed, while a set of proteins (at least five) ranging in size from 11 to 14 kDa was readily detectable. Murine antisera generated against SML-3 detected two proteins migrating at 16 and 18 kDa (Fig. 2C).

**Fig. 3:**
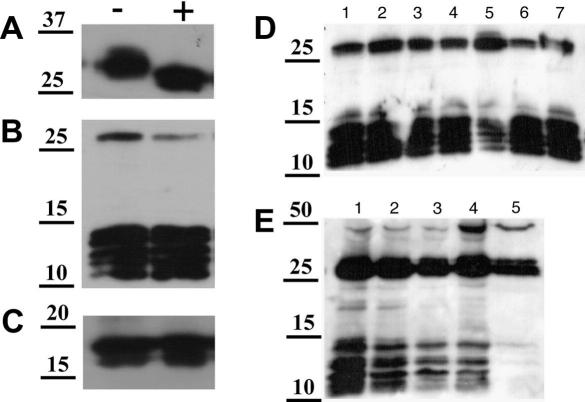
Analysis of post-translational modification and processing of secreted from muscle stage larvae (SMT) 1, 2 and 3. *Trichinella spiralis* muscle stage larvae secreted proteins (SPs) were treated with N-glycanase and probed by Western blot with antisera to SMLs: − no N-glycanase, + 50 U ml^−1^ N-glycanase. (A) Western blot probed with anti-SML-1. (B) Western blot probed with anti-SML-2. (C) Western blot probed with anti-SML-3. (D) The effects of a panel of protease inhibitors on post-translational processing of SML-2 was tested and a Western blot of treated SP probed with anti-SML-2 is shown. Lanes 1: no treatment, 2: 100 μM phenylmethylsulphonyl fluoride (PMSF), 3: 100 μM *N* α-Tosyl-Lys-chloromethylketone (TLCK), 4: 1 mM EDTA, 5: 100 μM 1,10-phenanthroline, 6: 1 μM pepstatin, 7: 1 mM *N*-ethylmaleimide (NEM). (E) The effects of increasing concentrations of 1,10-phenanthroline on processing of SML-2 was tested and a Western blot of treated SP probed with anti-SML-2 is shown. Lane 1: no treatment, 2: 1 μM, 3:10 μM, 4: 100 μM, 5: 1 mM 1,10-phenanthroline. Molecular mass markers are shown in kilodaltons.

Post-translational modification of native SMLs by N-linked glycosylation was tested by digesting mL1s SP with PNGase F. While SML-1 was observed to be glycosylated at a single site (Fig. 3A), neither SML-2 or SML-3 proteins showed any change in electrophoretic mobility (Fig. 3B and C).

Data shown in Fig. 2B and analysis of the SML-2 ORF suggested that the 11–14 kDa species detected in secreted products are derived from the larger primary products detected in somatic extracts. Proteolytic post-translational processing of SML-2 was tested using a panel of protease inhibitors. A specific inhibitor of zinc-dependent metalloproteases, 1,10-phenanthroline, blocked processing of SML-2 pro-proteins, with a reduction in the amounts of the lower molecular weight bands relative to the control and samples treated with other inhibitors (Fig. 3D). Neither E-64 nor aprotinin treatment had any effect on processing of SML-2 (data not shown). Subsequent titration of the activity of 1,10-phenanthroline demonstrated effects at concentrations as low as 10 μM, with almost complete loss of the lower molecular weight products in SP collected from mL1s cultured in the presence of 1 mM inhibitor (Fig. 3E).

### 3.3 Cellular localisation of native SMLs

**Fig. 4:**
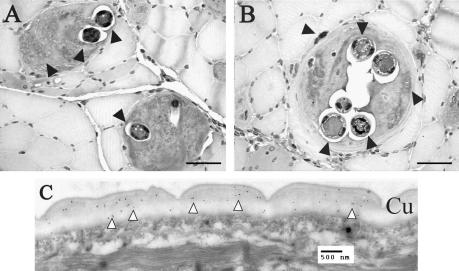
Histochemical and ultrastructural localisation of secreted from muscle stage larvae (SML)-1. Formalin-fixed paraffin embedded *Trichinella spiralis*-infected rat tissue 18 (A) or 28 (B) days p.i. probed with anti-SML-1 and detected with horseradish peroxidase-conjugated anti-IgG and 3,3′-diaminobenzidine substrate (dark grey and black colour). Sections were counterstained with Mayer’s haematoxylin (light grey colour). The black arrows show areas of staining either within the nematode or the nurse cell. Within the nematode staining can be observed within the hypodermal chord cells and haemolymph. Forty micrometres black scale bars are shown in both images. Transmission electron microscopy sections of *T. spiralis* mL1s were probed with anti-SML-1 (C) and visualised with goat anti-mouse IgG coupled to 15 nm gold particles (black dots). Localisation of SML-1 can be observed in the nematode cuticle (white arrows). A 500 nm scale bar is shown. Cu: nematode cuticle. A colour version of this figure, additional images and pre-bleed controls are shown in the Supplementary Figs. S1, S2 and S5.

**Fig. 5:**
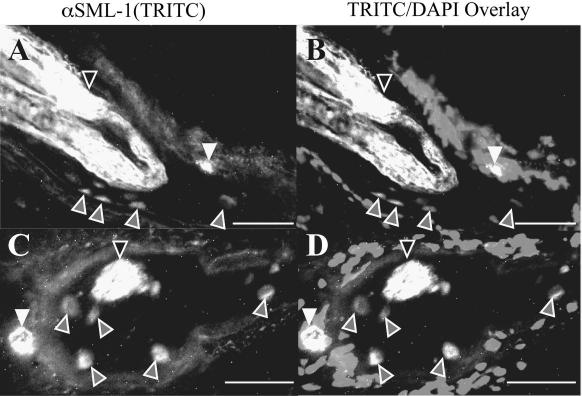
Ultrastructural localisation of secreted from muscle stage larvae (SML)-1 within nurse cells. Sections of methanol-fixed frozen *Trichinella spiralis* infected rat tissue (24 days p.i.) were probed with anti-SML-1 and binding visualised with tetramethyl rhodamine iso-thiocyanate (TRITC)-conjugated anti-IgG. (A) and (C) show TRITC labelling (white). Nuclei within the tissue were visualised with DAPI. (B) and (D) show the overlay of the DAPI (grey) and TRITC images (white). Localisation of SML-1 to the nematode (black arrows), the hypertrophic nuclei (grey arrows) and an unidentified structure on the edge of the nurse cell (white arrows) is highlighted. White scale bars = 40 μm. A colour version of this figure, additional images and pre-bleed controls are shown in the Supplementary Figs. S3, S4 and S14.

Three different techniques were used to localise native SMLs either within the nematode or *in situ* within infected muscle. Analysis of formalin-fixed/paraffin-embedded tissue and methanol-fixed frozen sections demonstrated that SML-1 was secreted from the parasites into the nurse cell. In the formalin-fixed material, immunoreactivity was observed in the hypertrophic nuclei and nurse cell cytoplasm (Fig. 4A and B). In the frozen sections, immunoreactivity was highest in the hypertrophic nuclei and an unidentified structure adjacent to or penetrating into the nurse cell capsule (Fig. 5). This structure can also be identified in formalin-fixed sections but is not as commonly observed as it is in the frozen material. Within the mL1s, anti-SML-1 reacted most strongly with hypodermal chord cells, haemolymph (Fig. 4A and B) or the cuticle (Fig. 5). Immunogold TEM localised native SML-1 within the cuticle, indicating it is associated with or translocated through this structure (Fig. 4C).

**Fig. 6:**
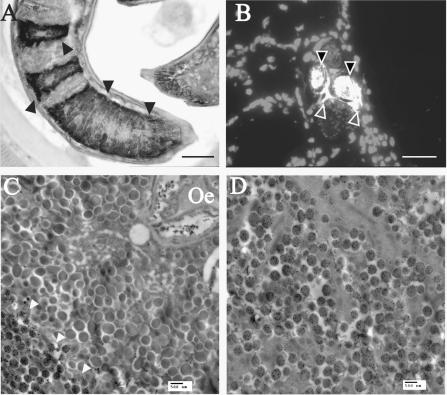
Ultrastructural localisation of secreted from muscle stage larvae (SML)-2. (A) Formalin-fixed paraffin embedded *Trichinella spiralis*-infected rat tissue 28 days p.i. probed with anti-SML-2 and detected with horseradish peroxidase-conjugated anti-IgG and 3,3′-diaminobenzidine substrate. Sections were counterstained with Mayer’s haematoxylin. The black arrows show areas of staining within the mL1s stichocytes. Sections of methanol-fixed frozen *T. spiralis*-infected rat tissue (24 days p.i.) were probed with anti-SML-2 and protein visualised with tetramethyl rhodamine iso-thiocyanate (TRITC)-conjugated anti-IgG. Nuclei within the tissue were visualised with DAPI. (B) Overlay of the TRITC (white) and DAPI (grey) images and localisation of SML-2 within the nematode (black arrows) and nurse cell cytoplasm (grey arrows). A 20 μm black (A) or 40 μm white (B) scale bar is shown in each image. Transmission electron microscopy sections of *T. spiralis* mL1s were probed with anti-SML-2 (C and D) and visualised with goat anti-mouse IgG coupled to 15 nm gold particles (black dots). Localisation of SML-2 to granules within the mL1s β-stichocytes (white arrows) can be observed. No staining of α-stichocyte granules was observed. Oe: Oesophagus. Scale bars (500 nm) are shown in each image. A colour version of this figure, additional images and pre-bleed controls are shown in the Supplementary Figs. S6–S9 and S14.

**Fig. 7:**
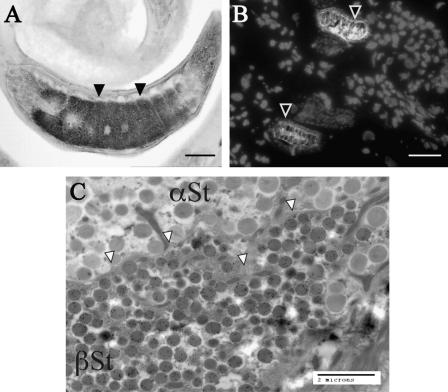
Ultrastructural localisation of secreted from muscle stage larvae (SML)-3. (A) Formalin-fixed paraffin embedded *Trichinella spiralis*-infected rat tissue 28 days p.i. probed with anti-SML-3 and detected with horseradish peroxidase-conjugated anti-IgG and 3,3′-diaminobenzidine substrate. Sections were counterstained with Mayer’s haematoxylin. The black arrows show areas of staining within the mL1s stichocytes. Sections of methanol-fixed frozen *T. spiralis* infected rat tissue (24 days p.i.) were probed with anti-SML-3 and protein visualised with tetramethyl rhodamine iso-thiocyanate (TRITC)-conjugated anti-IgG. Nuclei within the tissue were visualised with DAPI. (B) Overlay of a TRITC (white) and DAPI (grey) images and localisation of SML-3 within the nematode (black arrows) but not the nurse cell. A 20 μm black (A) or 40 μm white (B) scale bar is shown in each image. Transmission electron microscopy sections of *T. spiralis* mL1s were probed with anti-SML-3 (C) and visualised with goat anti-mouse IgG coupled to 15 nm gold particles (black dots). Localisation of SML-3 to granules within the mL1s β-stichocytes (white arrows) can be observed. No staining of α-stichocyte granules was observed. A 2 μm is shown. αSt: α-stichocyte granules and βSt: β-stichocyte granules. A colour version of this figure, additional images and pre-bleed controls are shown in the Supplementary Figs. S10–S14.

Both SML-2 and -3 were localised to the mL1s stichosome (Fig. 6A and 7A), and this was confirmed by immunogold TEM, which showed both proteins in dark granules within the β-stichocytes (Figs. 6C, D and 7C). While we were unable to localise either protein outside the parasite in formalin-fixed tissue, SML-2 could be detected within the nurse cell cytoplasm surrounding the parasite in frozen sections (Fig. 6B).

**Supplementary Fig. S1:**
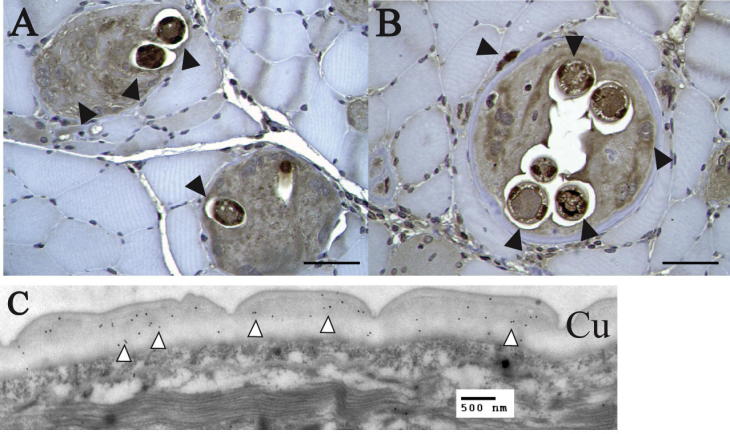
Histochemical and ultrastructural localisation of secreted from muscle stage larvae (SML)-1. This figure shows a colour version of Fig. 4. Formalin-fixed paraffin embedded *Trichinella spiralis*-infected rat tissue 18 (A) or 28 (B) days p.i. probed with anti-SML-1 and detected with horseradish peroxidase-conjugated anti-IgG and 3,3′-diaminobenzidine substrate (brown colour). Sections were counterstained with Mayer’s haematoxylin (purple colour). The black arrows show areas of staining either within the nematode or the nurse cell. Within the nematode staining can be observed within the hypodermal chord cells and haemolymph. Forty micrometres black scale bars are shown in each image. Transmission electron microscopy sections of *T. spiralis* mL1s were probed with anti-SML-1 (C) and visualised with goat anti-mouse IgG coupled to 15 nm gold particles (black dots). Localisation of SML-1 can be observed in the nematode cuticle (white arrows). A scale bar in nm is shown. Cu: nematode cuticle. Additional images and pre-bleed controls are shown in the Supplementary Figs. S2 and S5.

**Supplementary Fig. S2:**
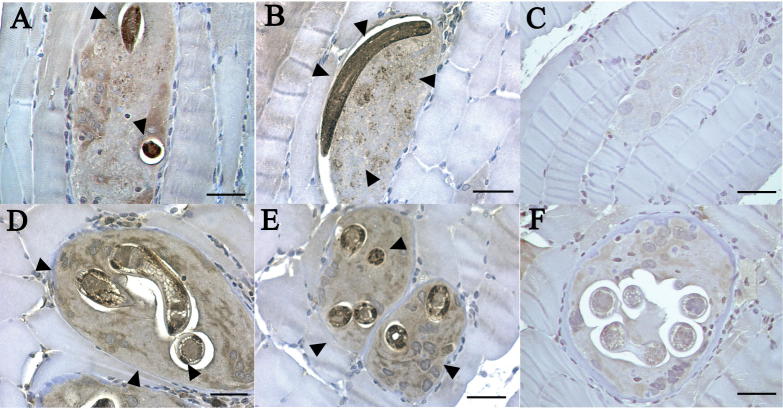
Immunohistochemical analysis of secreted from muscle stage larvae (SML)-1. *Trichinella spiralis* infected rat muscle was collected 18 (A, B and C) or 28 (D, E and F) days p.i., fixed in formalin and embedded in paraffin. Thin sections of infected muscle were probed with anti-SML-1 (A, B, D and E) or anti-SML-1 pre-bleed control sera (C and F) and detected with horseradish peroxidase-conjugated anti-IgG and 3,3′-diaminobenzidine substrate (brown colour). Sections were counterstained with Mayer’s haematoxylin (purple colour). The black arrows show areas of staining either within the nematode or the nurse cell. Within the nematode staining can be observed within the cuticle, hypodermal chord cells and haemolymph. Scale bars = 40 μm.

**Supplementary Fig. S3:**
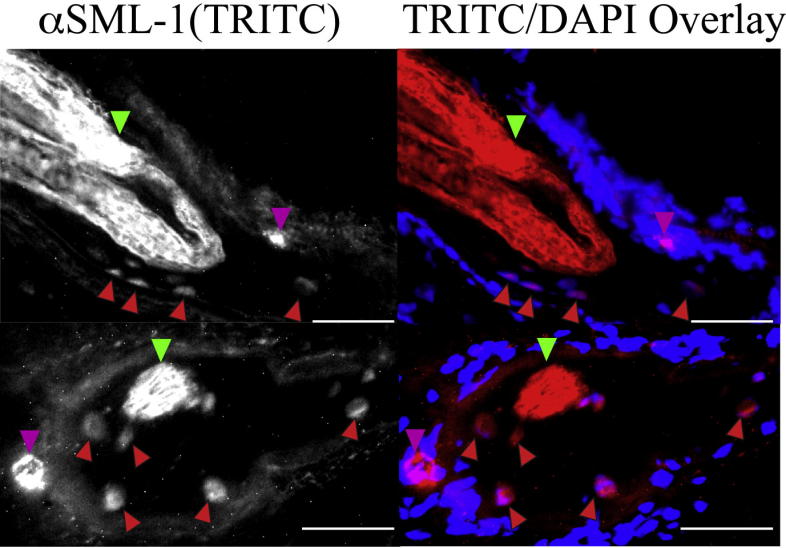
Ultrastructural localisation of secreted from muscle stage larvae (SML)-1 within nurse cells. This figure shows a colour version of Fig. 5. Sections of methanol-fixed frozen *Trichinella spiralis* infected rat tissue (24 days p.i.) were probed with anti-SML-1 and protein visualised with tetramethyl rhodamine iso-thiocyanate (TRITC)-conjugated anti-IgG. The left hand panel shows TRITC labelling (black and white). Nuclei within the tissue were visualised with DAPI. The right hand panel shows a colour overlay of the DAPI (blue) and TRITC images (red). Localisation of SMLs to the nematode (green arrows), the hypertrophic nuclei (red arrows) and an unidentified structure on the edge of the nurse cell (purple arrows) is highlighted. Forty micrometres white scale bars are shown in each image. Additional images, single channel images and pre-bleed controls are shown in Supplementary Figs. S4 and S14.

**Supplementary Fig. S4:**
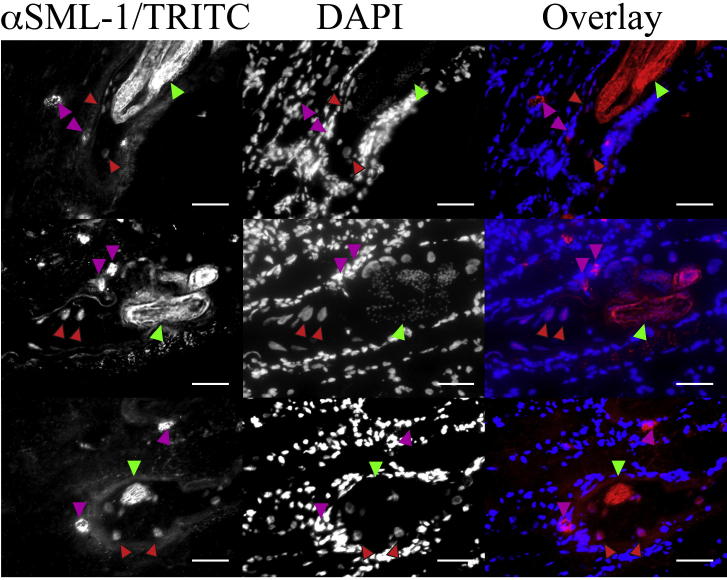
Immunofluorescence analysis of secreted from muscle stage larvae (SML)-1. Frozen sections were cut from *Trichinella spiralis* infected rat tissue collected 24 days p.i., fixed in methanol and then probed with anti-SML-1. SML-1 protein was visualised with tetramethyl rhodamine iso-thiocyanate (TRITC)-conjugated anti-mouse IgG (red). Nuclei within the tissue were visualised with DAPI (blue). The single channel (black and white) and an overlay image (colour) are shown. Localisation to the nematode (green arrows), nurse cell hypertrophic nuclei (red arrows) and an unidentified structure on the edge of the nurse cell (purple arrows) are highlighted. Pre-bleed controls are shown in Supplementary Fig. S14. Scale bars = 40 μm.

**Supplementary Fig. S5:**
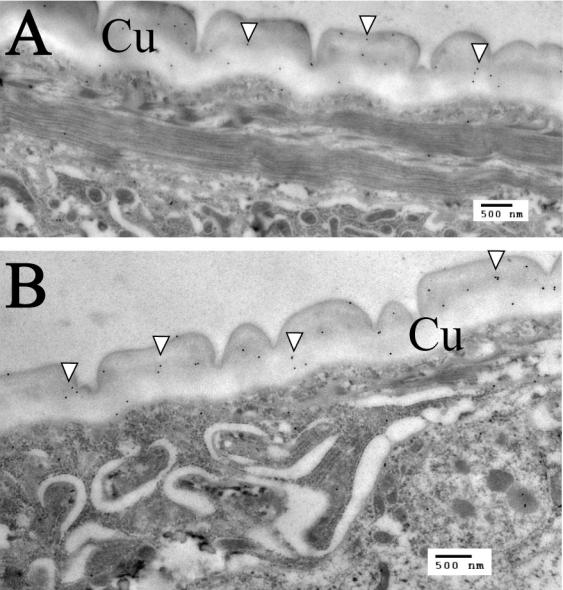
Structural localisation of secreted from muscle stage larvae (SML)-1 within *Trichinella spiralis* mL1s. Thin sections of *T. spiralis* mL1s were incubated with anti-SML-1 antibodies, followed by incubation with goat anti-mouse IgG coupled to 15 nm gold particles (black dots) for ultrastructural localisation of the native SML-1 (A and B). Localisation of SML-1 can be observed in the nematode cuticle (Cu, white arrows). Scale bars = 500 nm.

**Supplementary Fig. S6:**
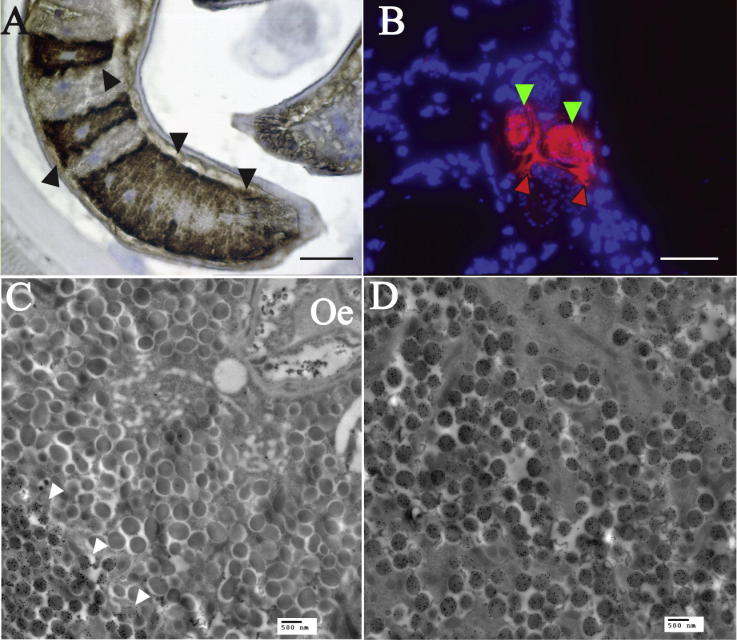
Ultrastructural localisation of secreted from muscle stage larvae (SML)-2. This figure shows a colour version of Fig. 6. (A) Formalin-fixed paraffin embedded *Trichinella spiralis*-infected rat tissue 28 days p.i. probed with anti-SML-2 and detected with horseradish peroxidase-conjugated anti-IgG and 3,3′-diaminobenzidine substrate. Sections were counterstained with Mayer’s haematoxylin. The black arrows show areas of staining within the mL1s stichocytes. Sections of methanol-fixed frozen *T. spiralis* infected rat tissue (24 days p.i.) were probed with anti-SML-2 and protein visualised with tetramethyl rhodamine iso-thiocyanate (TRITC)-conjugated anti-IgG. Nuclei within the tissue were visualised with DAPI. (B) Overlay of the TRITC (red) and DAPI (blue) images and localisation of SML-2 within the nematode (green arrows) and nurse cell cytoplasm (red arrows). A 20 mm black (A) or 40 mm white (B) scale bar is shown in each image. Transmission electron microscopy sections of *T. spiralis* mL1s were probed with anti-SML-2 (C and D) and visualised with goat anti-mouse IgG coupled to 15 nm gold particles (black dots). Localisation of SML-2 to granules within the mL1s β-stichocytes (white arrows) can be observed. No staining of α-stichocyte granules was observed. Oe: Oesophagus. Scale bars in nm are shown in each image. Additional images, single channel images and pre-bleed controls are shown in Supplementary Figs. S7, S8, S9 and S14.

**Supplementary Fig. S7:**
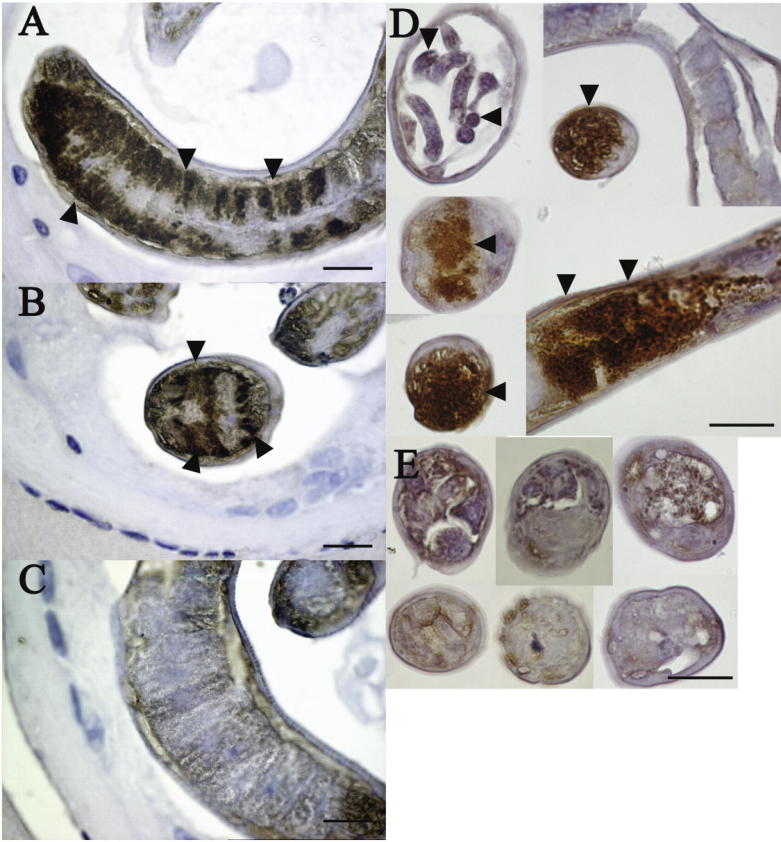
Immunohistochemical analysis of secreted from muscle stage larvae (SML)-2. SML-2 protein was localised in formalin-fixed, paraffin embedded *Trichinella spiralis*-infected rat tissue collected 28 days p.i. (A–C) Thin sections were probed with anti-SML-2 (A and B) or anti-SML-2 pre-bleed control sera (C) and detected with horseradish peroxidase-conjugated anti-IgG and 3,3′-diaminobenzidine substrate. Sections were counterstained with Mayer’s haematoxylin. The black arrows show areas of intense staining within the nematode. Localisation of SML-2 in adults and uterine NBLs was also examined. *Trichinella spiralis* adults were isolated 6 days p.i., formalin-fixed and paraffin embedded. Thin sections were cut to poly-lysine coated slides and probed with anti-SML-2 (D) or SML-2 (E) pre-bleed control sera. Black arrows indicate areas of cross reactivity either in the stichocytes of the adults or small vesicles in the uterine NBLs (D) or equivalent anatomical sections probed with pre-bleed sera (E). Scale bars = 20 μm.

**Supplementary Fig. S8:**
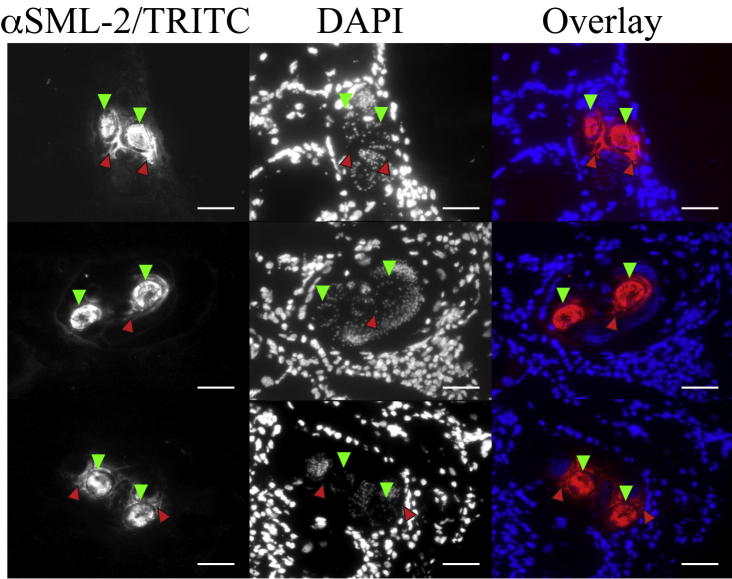
Immunofluorescence analysis of secreted from muscle stage larvae (SML)-2/MCD-1. Frozen sections were cut from *Trichinella spiralis*-infected rat tissue collected 24 days p.i., fixed in methanol and then probed with anti-SML-2. SML-2 protein was visualised with tetramethyl rhodamine iso-thiocyanate (TRITC)-conjugated anti-mouse IgG (red). Nuclei within the tissue were visualised with DAPI (blue). The single channel (black and white) and an overlay image (colour) are shown. Localisation to the nematode (green arrows) and the nurse cell cytoplasm (red arrows) are highlighted. Pre-bleed controls are shown in Supplementary Fig. S14. Scale bars = 40 μm.

**Supplementary Fig. S9:**
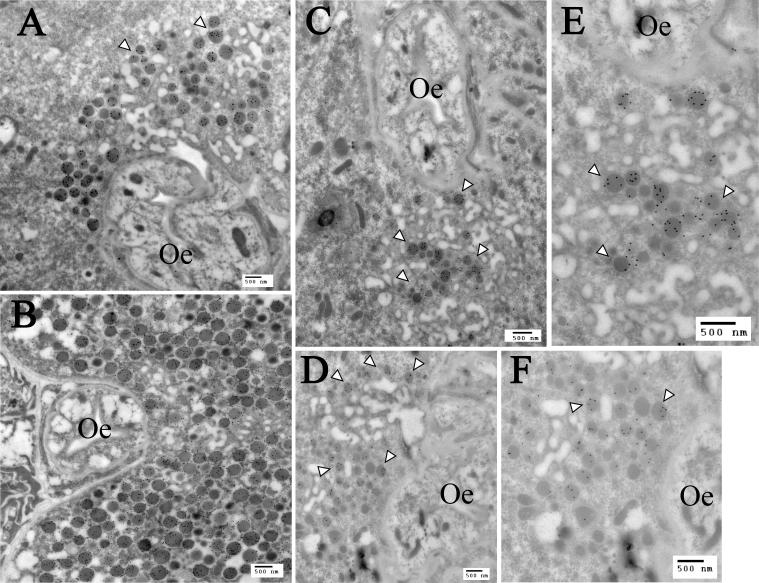
Structural localisation of secreted from muscle stage larvae (SML)-2 within different developmental stages of *Trichinella spiralis*. For ultrastructural localisation of the native SML-2 thin sections of *T. spiralis* adults and NBL were incubated with anti-SML-2 antibodies, followed by incubation with goat anti-mouse IgG coupled to 15 nm gold particles (black dots) (A–D). Localisation of SML-2 can be observed in the stichocyte granules of the adults (A and B) as well as secretory vesicles with in the NBL (C and D). (E) and (F) shows a magnified view of images (C) and (D). Scale bars = 500 nm. Oe: Oesophagus.

**Supplementary Fig. S10:**
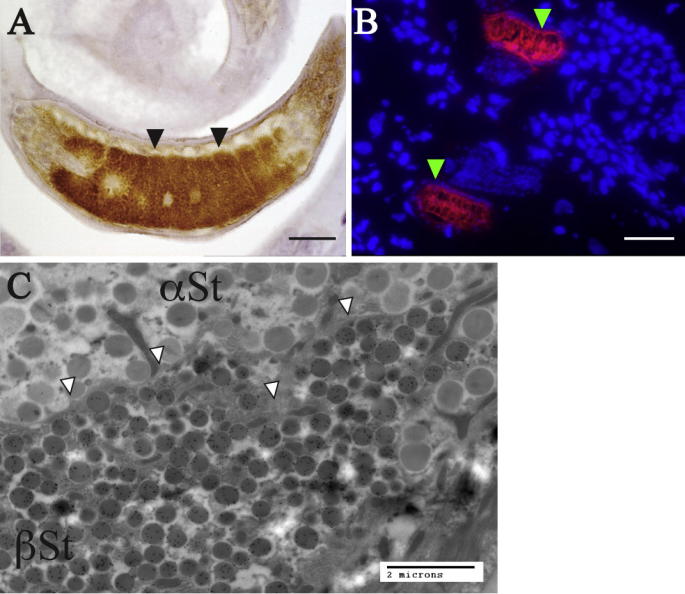
Ultrastructural localisation of secreted from muscle stage larvae (SML)-3. This figure shows a colour version of Fig. 7. (A) Formalin-fixed paraffin embedded *Trichinella spiralis*-infected rat tissue 28 days p.i. probed with anti-SML-3 and detected with horseradish peroxidase-conjugated anti-IgG and 3,3′-diaminobenzidine substrate. Sections were counterstained with Mayer’s haematoxylin. The black arrows show areas of staining within the mL1s stichocytes. Sections of methanol-fixed frozen *T. spiralis* infected rat tissue (24 days p.i.) were probed with anti-SML-3 and protein visualised with tetramethyl rhodamine iso-thiocyanate (TRITC)-conjugated anti-IgG. Nuclei within the tissue were visualised with DAPI. (B) Overlay of a TRITC (red) and DAPI (blue) images and localisation of SML-3 within the nematode (green arrows) but not the nurse cell. A 20 μm black (A) or 40 μm white (B) scale bar is shown in each image. Transmission electron microscopy sections of *T. spiralis* mL1s were probed with anti-SML-3 (C) and visualised with goat anti-mouse IgG coupled to 15 nm gold particles (black dots). Localisation of SML-3 to granules within the mL1s β-stichocytes (white arrows) can be observed. No staining of α-stichocyte granules was observed. Scale bar = 2 μm. αSt: α-stichocyte granules and βSt: β-stichocyte granules. Additional images, single channel images and pre-bleed controls are shown in Supplementary Figs. S11–S14.

**Supplementary Fig. S11:**
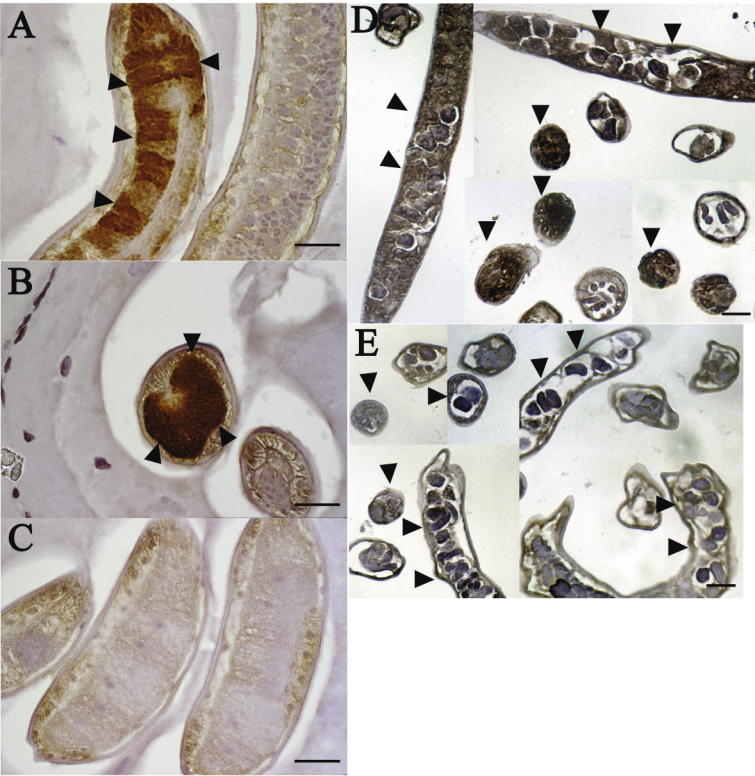
Immunohistochemical analysis of secreted from muscle stage larvae (SML)-3. SML-3 protein was localised in formalin-fixed, paraffin embedded *Trichinella spiralis* infected rat tissue collected 28 days p.i. (A–C) Thin sections were probed with anti-SML-3 (A and B) or anti-SML-3 pre-bleed control sera. (C). The black arrows show areas of intense staining within the nematode. Localisation of SML-3 in adults and developing embryos was also examined. *Trichinella spiralis* adults were isolated 6 days p.i., formalin-fixed and paraffin embedded. Thin sections were cut to poly-lysine coated slides and probed with anti-SML-3 (D) or SML-3 pre-bleed control sera (E). Black arrows indicate areas of cross reactivity either in the stichocytes and body wall of the adults or developing embryos in utero (D) or equivalent anatomical sections probed with pre-bleed sera (E). Scale bars = 20 μm.

**Supplementary Fig. S12:**
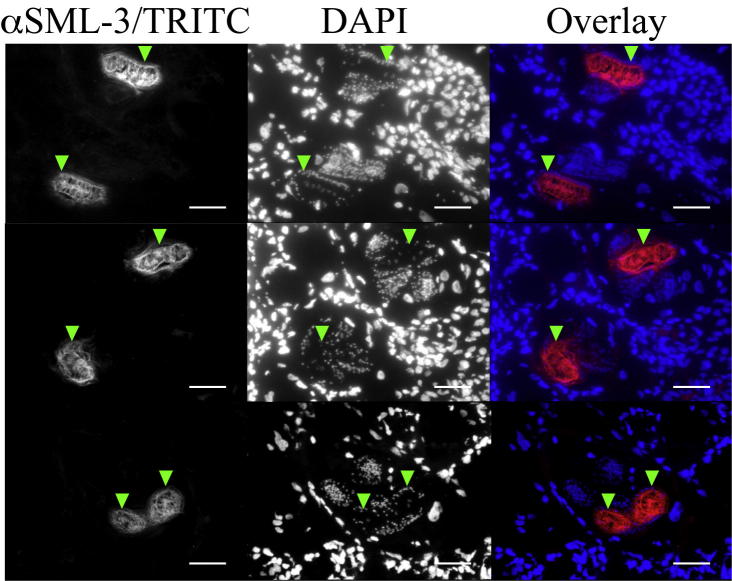
Immunofluorescence analysis of secreted from muscle stage larvae (SML)-3. Frozen sections were cut from *Trichinella spiralis* infected rat tissue collected 24 days p.i., fixed in methanol and then probed with anti-SML-3. SML-3 protein was visualised with tetramethyl rhodamine iso-thiocyanate (TRITC)-conjugated anti-mouse IgG (red). Nuclei within the tissue were visualised with DAPI (blue). The single channel (black and white) and an overlay images (colour) are shown. Localisation to the nematode (green arrows) but not the nurse cell is highlighted. Pre-bleed controls are shown in Supplementary Fig. S14. Scale bars = 40 μm.

**Supplementary Fig. S13:**
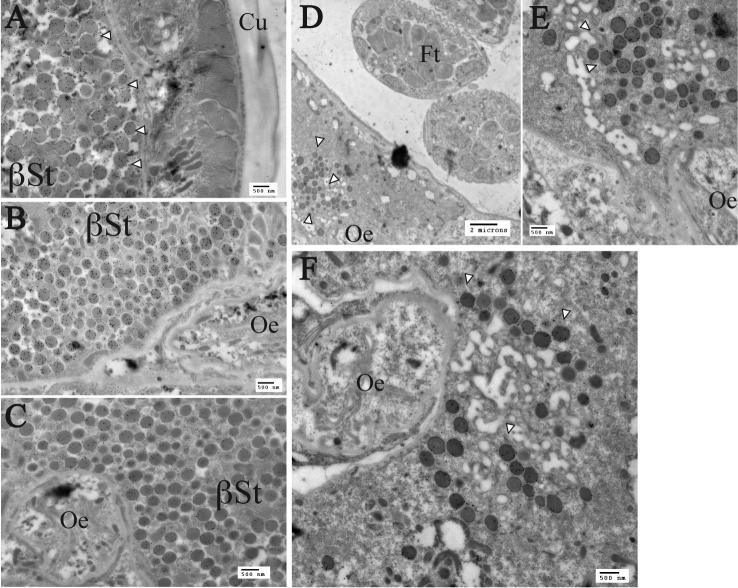
Structural localisation of secreted from muscle stage larvae (SML)-3 within the stichocytes of different developmental stages of *Trichinella spiralis*. For ultrastructural localisation of the native SML-3 thin sections of *T. spiralis* mL1s and adults were incubated with anti-SML-3 antibodies, followed by incubation with goat anti-mouse IgG coupled to 15 nm gold particles (black dots) (A–F). Localisation of SML-3 can be observed within the granules of mL1s β-stichocytes (A–C), stichosome of the adults (F) and granules within posterior stichocyte adjacent to the vagina in adult females (D and E). (E) Magnified view of image (D). βSt: β-stichocyte granules, Ft: Foetus, Oe: Oesophagus. 500 nm scale bars are shown in images (A–C) and (E–F) while a 2 μm scale bar is shown in image (D).

**Supplementary Fig. S14:**
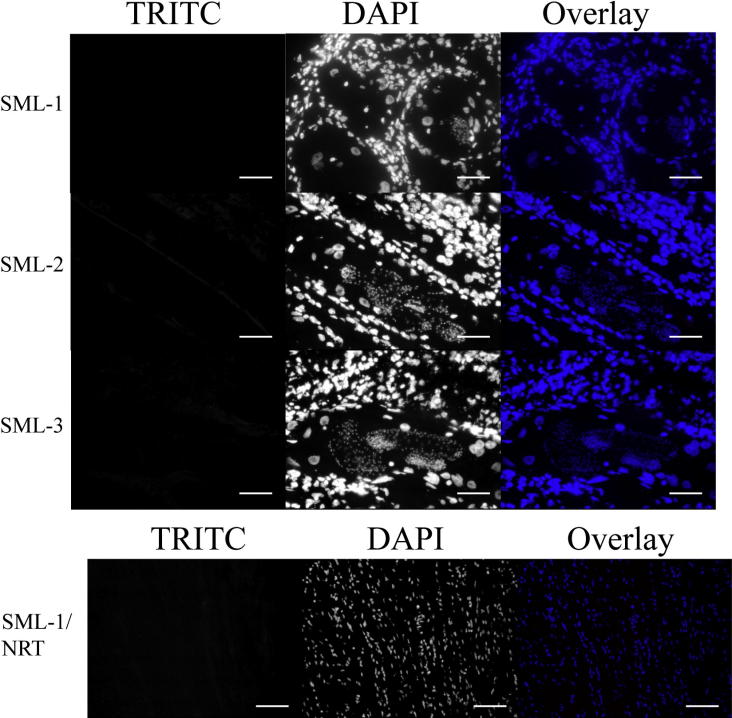
Secreted from muscle stage larvae (SML)-1, 2 and 3 immunofluorescence pre-bleed controls. (A) Frozen sections were cut from *Trichinella spiralis* infected rat tissue collected 24 days p.i., fixed in methanol and then probed with pre-bleed sera collected from the groups of mice prior to immunisation with SMLs-1, -2, and -3. Cross-reactive antibodies were visualised with tetramethyl rhodamine iso-thiocyanate (TRITC)-conjugated anti-mouse IgG (red). Nuclei within the tissue were visualised with DAPI (blue). The single channel (black and white) and an overlay image (colour) are shown. No cross-reactivity in the infected muscle can be observed with any of the pre-bleed anti-sera. (B) SML-1 immunofluorescence in control uninfected muscle. Frozen sections were cut from uninfected normal rat muscle (NRT), fixed in methanol and then probed with anti-SML-1 sera. Cross-reactive antibodies were visualised with TRITC-conjugated anti-mouse IgG (red). Nuclei within the tissue were visualised with DAPI (blue). The single channel (black and white) and an overlay image (colour) are shown. No cross reactivity in the normal muscle can be observed with anti-SML-1 sera. Scale bar = 40 μm.

Colour versions of immunohistochemical and immunofluorescence images found in Figs. 4–7 are shown in Supplementary Figs. S1, S3, S6 and S10. Localisation of each protein is shown in more detail in the Supplementary Figs. S2, S4, S5, S7, S8, S9, S11–S14 which also highlight the lack of reactivity of each antiserum to surrounding muscle fibres and infiltrating mononuclear cells, and the lack of reactivity of pre-bleed control sera to parasites and infected host tissue. We also examined other stages of the parasite and observed that SML-2 was localised to granules within the adult stichosome and granules within NBL (Supplementary Fig. S9). SML-3 was found to have an overlapping localisation pattern in mL1s and adults but was not detected in NBLs. In addition SML-3 was identified in granules in the posterior stichocytes of adult females adjacent to the vagina (Supplementary Fig. S13).

### 3.4 Identification and analysis of multiple members of the *sml-2/mcd-1* and *sml-3* gene families

**Fig. 8:**
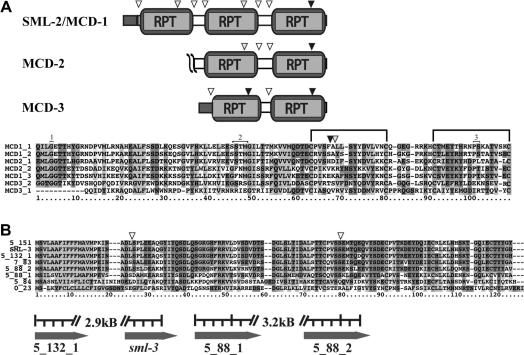
Analysis of secreted from muscle stage larvae (SML)-2/multi-domain ‘cystatin-like’ protein (MCD)-1 and 3 gene families. Comparison of *sml* genes to the *Trichinella spiralis* genome using BLAST identified MCD-2, MCD-3 and a family of SML-3-like genes. (A) A second gene with high levels of similarity to SML-2/MCD-1 was identified in the *T. spiralis* genome and EST datasets. MCD-3 was recently deposited in GenBank by Liu et al. (ABY59464). Only two ‘cystatin-like’ domains can be identified in the *mcd-2* gene prediction or the confirmed *mcd-3* sequence. The intron positions within the *mcds* are indicated with triangles. Introns outside the repeated domains (white) and a conserved intron occurring four (black) or five (light grey) amino acids after the first cysteine residue are shown. The cystatin-like domains contained in SML-2/MCD-1 (1–3), MCD-2 (1–2), and MCD-3 (1–2) were aligned with ClustalX and demonstrate high levels of sequence conservation. The positions of the conserved N-terminal glycine (1), degenerate Q-x-V-x-G motif (2), PW motif (3), cysteine residues (linked bars), and intron positions (light grey or black triangle) are shown. (B) The predicted protein sequences of *sml-3* gene family members were aligned with ClustalX. The two intron positions, which are conserved in all the *sml-3*-like genes, are shown with white triangles. Two pairs of the *sml-3*-like sequences sharing common transcriptional orientations are found in close apposition (∼3 kB distance) on *T. spiralis* genomic contigs 5_132 and 5_88. The sequences of gene predictions used in this analysis are listed in Supplementary Table S2.

Analysis of the EST and genome datasets identified a second gene closely related to *sml-2*/*mcd-1* (ps46b06.y1, BG521250, NEMBASE cluster numbers TSC0210 and TSC1941), and we have designated this gene *mcd-2* (Fig. 8A). Like the *mcd-3* sequence submitted to GenBank (Accession Number ABY59464) by Liu and colleagues, the predicted protein encoded by *mcd-2* contains only two ‘cystatin-like’ repeats. While the ESTs represent a partial cDNA and the sequence of this gene has not been independently confirmed, we were unable to identify any additional ‘cystatin-like’ repeats in this genomic region. Comparisons of the *mcd* family domain architectures, intron positions, and sequence conservation within the repeated domains are shown in Fig. 8A. There is a high degree of sequence conservation between the individual domains (38–80% similarity) and many of the ‘cystatin-like motifs’ identified by Robinson are also conserved, as are the N-terminal glycine (with the exception of the first domain in MCD-3), four cysteines, and a glycine residue within the degenerate Q-x-V-x-G motif (Robinson et al., 2007). Analysis of sequence synaptomorphies and conserved intron positions within these genes indicates that the second domain in SML-2/MCD-1 may have arisen by duplication of the first domain in an ancestral *mcd* gene (Fig. 8A).

A similar analysis indicated that *sml-3* is part of a large gene family (at least eight members) of small putatively secreted proteins. Fig. 8B shows an alignment of *sml-3* and the additional gene family members. Overall sequence conservation between these genes is very high (50–98% similarity), including four cysteine residues potentially involved in disulphide bond formation and two conserved intron sites. Two pairs of these genes (5_132_1, *sml-3* and 5_88_1, 5_88_2) are found in close apposition (∼3 kB intergenic distances) on *T. spiralis* genomic contigs and share common transcriptional orientations, indicating that some members of this gene family may have arisen from short-range duplication events.

## 4 Discussion

We have utilised an informatics analysis of the clustered *T. spiralis* EST datasets to identify a number of candidate molecules that could be secreted into the nurse cell, and present the characterisation of three of the top candidates. At the time of the analysis three of these candidates which we had shown were secreted by mL1s had no significant homology to any characterised sequences in GenBank, were given the designation secreted by muscle stage larvae (SMLs). *sml-1* is specifically expressed in mL1s, while *sml-2* and *-3* are expressed in all parasite stages analysed. All three SMLs are secreted by mL1s, and SML-2 and -3 are also secreted by adult parasites. Moreover, immunohistochemical analysis suggests that SML-1 and -2 are secreted into the nurse cell, with SML-1 localised primarily to hypertrophic nuclei while SML-2 is contained in the host cytoplasm surrounding the parasite.

Use of the sub-cellular localisation algorithm PSORT indicates that SML-1 could be a nuclear protein, and immunohistochemical staining supports this possibility. In addition, SML-1 was also localised to structures adjacent to the nurse cell capsule. We have not been able to definitively identify these structures, although their morphology and location is indicative of blood vessels that form the circulatory rete around the nurse cell. Within the parasite, SML-1 was localised to multiple structures including the cuticle, hypodermal chord cells and haemolymph. Secretion of this molecule may therefore be trans-cuticular rather than via the stichosomes. Trans-cuticular secretion has been observed with other nematode proteins (Selkirk et al., 1990) and *T. spiralis*-secreted TSJ5 may be exported via this route (Kuratli et al., 2001).

SML-2 and -3 have also been identified in larval secreted products by proteomics (Robinson and Connolly, 2005; Robinson et al., 2007). SML-2/MCD-1 resolved as 47, 35/36 and 32/33 kDa species by SDS–PAGE. Heterologous expression in HeLa cells established that the 47 kDa protein was processed in a pH dependent autocatalytic manner into the 33 kDa species via removal of the C-terminal ‘cystatin-like’ domain. We have independently verified the processing of SML-2, detecting both the 47 and 32/33 kDa species in our SP preparations with an antibody probe. We have also demonstrated that these polypeptides are further proteolytically processed further into a set of peptides spanning 11–14 kDa, masses which correspond to the predicted size of monomeric cystatin domains. The heterogeneity of these smaller protein species is not due to N-linked glycosylation, suggesting that the processing events might occur at variable cleavage sites. Inhibition of processing by 1,10-phenanthroline implicates a zinc-dependent metalloprotease, and this class of enzyme has been identified in secreted products from *T. spiralis* larvae (Lun et al., 2003). Metalloproteases are involved in a number of biological processes in nematodes including moulting, hormone processing and tissue invasion, and we have established that processing of secreted proteins can be added to this list. A recent proteomic analysis of mL1s SP failed to identify these lower molecular weight species (Robinson and Connolly, 2005). Both our data and that presented by Robinson et al. (2005, 2007) suggest that processing of SML-2/MCD-1 in SP is progressive with longer culturing time yielding more fully processed species. As our SP was collected from mL1s cultured for almost twice the amount time as used in their study this would have substantially changed the composition of the SML-2/MCD-1 isoforms. In addition this survey did not include many lower molecular weight components of mL1s SP and the smallest protein identified migrated with an apparent molecular weight of 15 kDa. Thus, it seems likely the incongruities between datasets are likely to have arisen from a number of methodological differences.

We localised SML-2/MCD-1 to granules within the stichosome in mL1s and adult parasites, suggesting it is synthesised and secreted from this organ. In NBLs the protein was found in vesicles of cells surrounding the oesophagus, suggesting it is also secreted by this stage. In contrast to SML-1, secretion of SML-2 into the nurse cell cytoplasm could only be observed in sections of methanol-fixed frozen tissue. SML-3 is secreted by both larvae and adult worms, and was detectable by Western blot as two bands at 16 and 18 kDa, some 6–8 kDa larger than the predicted mass. This is not due to N-linked glycosylation, suggesting that it undergoes an alternative form of post-translational modification or migrates anomalously in SDS–PAGE. Like SML-2, SML-3 is stored in *T. spiralis* stichosome granules, but immunohistochemical analysis indicates it is also found in the uterine lining and on the surface of embryos in adult female nematodes (Supplementary Fig. S13).

We identified a gene encoding a protein with two ‘cystatin-like’ domains, closely related to those found in SML-2/MCD-1 (55–80% peptide similarity in domain sequences), and have designated this *mcd-2*. We also identified a family of genes (at least eight) encoding proteins that are closely related to *sml-3* (50–98% peptide sequence similarity). The high degree of sequence similarity may explain the multiple bands detected by Western blotting observed in this study and the complex isoform profiles observed by proteomics (Robinson and Connolly, 2005). In addition to a number of other characterised proteins and enzymatic activities (Ts53, prosaposin, nucleoside diphosphate kinase, hexosaminidase and GM2 activator) neither SML-1 or the smaller processed peptides of SML-2 were identified in the Robinson proteomic analysis (Zarlenga and Gamble, 1990; Gounaris et al., 2001; Selkirk et al., 2004; Robinson and Connolly, 2005; Bruce et al., 2006; Bruce and Gounaris, 2006). Thus, our informatic analysis has yielded a number of additional candidate molecules, and it is likely that a combination of methodologies will be required to fully characterise all the component proteins found in *T. spiralis* secretions.

SML-1 is a novel protein with significant similarity only to sequences from other Trichinellids, and thus any functional inferences are purely speculative. However, its localisation to the hypertrophic nuclei of nurse cells during their later stages of development suggests that it may play a role in either maintaining cell cycle dysregulation or initiating its novel transcriptional programme. We are currently performing in vitro functional screens to determine the effects of ectopic expression of SML-1 on the cell cycle status or transcriptional profiles of skeletal muscle cells.

SML-2/MCD-1 has weak homology to ‘cystatin-like’ protease inhibitors, but lacks a number of critical residues required for activity. Its localisation within the nurse cell cytoplasm around the parasite suggests that it may be interacting with other proteins/factors in this location. Robinson et al. (2007) could not demonstrate protease inhibitory activity, using bacterially expressed recombinant MCD-1. However, the recombinant protein used in their study contained all three ‘cystatin-like’ domains and was assayed only against papain. Thus, it is possible that SML-2/MCD-1’s activity is dependent upon correct folding and complete processing or may be specific for other cysteine proteases (Robinson et al., 2007). We are currently expressing the individual domains of the SML-2/MCD-1 in *Pichia pastoris* and will test the inhibitory properties of these peptides on a wider panel of proteases.

The analysis reported here has identified a number of secreted proteins which are likely to be involved in the intracellular survival of *T. spiralis*, and some of which may play a role in muscle fibre remodelling. Elucidation of the mechanisms involved in transformation of skeletal muscle will require the establishment of a robust map of the molecular events which underlie nurse cell genesis, further identification of parasite-secreted proteins and the development of assay systems for analysis of their effects on host cell function.

## Figures and Tables

**Table 1:** Candidates isolated from *Trichinella spiralis* muscle stage larvae (mL1s) expressed sequence tag (EST) datasets.

Name/Identifier	mL1	Adult	Homologies and EST cluster numbers
*grn-1*	17	–	Granulin domains (TSC00180)
*sml-1*	17	–	Novel (TSC00585, TSC02174, TSC01706)
*gp45*	15	21	Similar to serine proteinases (TSC00054, TSC03042, TSC01842) (Arasu et al., 1994)
9.10/*mcd-1*/*sml-2*	12	62	Contains three domains with weak similarity to type II cystatins (TSC01154, TSC00403, TSC03060, TSC03972, TSC05407, TSC03642, TSC03713, TSC02468, TSC03015) (Robinson et al., 2007)
11.3/*sml-3*	8	8	Novel (TSC00935, TSC00437)

Shown are the top five candidates identified in the informatics screen of the *T. spiralis* mL1s EST dataset. The columns next to the identifier show the number of ESTs for each transcript that were identified in the *T. spiralis* mL1s and adult EST datasets. Homologies to sequences contained within GenBank and the NEMBASE cluster numbers from which the ESTs are derived are also shown (Parkinson et al., 2004. No NBL ESTs were members of the clusters derived from these genes.
