# Supplementary material for: Characterisation of novel protein families secreted by muscle stage larvae of Trichinella spiralis
Source: Int J Parasitol. 2009 Apr;39(5):515–24. doi: 10.1016/j.ijpara.2008.09.012 (PMC2680962; doi:10.1016/j.ijpara.2008.09.012)
Supplement: Supplementary data [file mmc1.doc]

**Supplementary Table S1. Primer sequences used in this study.**

| Primer name | Primer Sequence | Description |
| --- | --- | --- |
| 38e09.pET29b.F1 | CATATGAAATTCGAATGCAATGACGAA | SML-1, RT-PCR and pET29b cloning primer |
| 38e09.pET29b.R1 | CTCGAGTTTGCATACTCTTGACATTTC | SML-1, RT-PCR pET29b cloning primer |
| 94b11.pET29b.F1 | CATATGCAGATACTTGGCGAAACA | SML-2, RT-PCR and pET29b cloning primer |
| 94b11.pET29b.R1 | CTCGAGACATTCAACAGTTGACTT | SML-2, RT-PCR and pET29b cloning primer |
| 32b06.pET29b.F1 | CATATGGATTTGAGTCCATTGGAAGAA | SML-3, RT-PCR and pET29b cloning primer |
| 32b06.pET29b.R1 | CTCGAGATGCCCATAATATGTACATTC | SML-3, RT-PCR and pET29b cloning primer |
| Ts-TBA-1.F1 | GGTCACATATGCACCGGTGAT | *Trichinella spiralis* alpha tubulin RT-PCR primer |
| Ts-TBA-1.R1 | GCTGTTGTATTAGATAACATGC | *T. spiralis* alpha tubulin RT-PCR primer |

The names, sequences, and descriptions of uses of primers utilized in this study are listed.

**Supplementary Table S2.** Secreted from muscle stage larvae (*sml)-2/* multi-domain ‘cystatin-like’ protein (*mdc*)-1 and *-3* gene family members identified in the *Trichinella spiralis* genome

| Gene Identifier | Predicted cDNA sequence | Predicted peptide sequence | EST clones | Genbank Accession | NEMBASE Cluster number |
| --- | --- | --- | --- | --- | --- |
| *mcd-2* | ATTGAGATGCTTGGTGGAACAACCCTTCATGGTAGAGATGCTGCTGTTATGTTCCCAGAAGCTCAGGAGGCATTATTCTTATCGGACACAAAACATAAGTTGGGTGTATTTCACATTCTGTTAAGAGTGGAAGAAGTTTCAACCATGGGAATCTTTACCATCATGCAAGTCGTTCTACAAAATACTGATTGCCAAGTTTCACATGACGTATCTTCCTATAACGATGTACTAAAAAAGTGCCGAGCTGAAAGTGAAAAACAGAAGTGTAGAATCGAATATAAGTATCATGATCCTTTAACAGCAACTGCCCTTTGTGTAAAGAAAGTAGAAGAATCGATAATTATACCGCAACGTGACCAAAGGGCGAATAGAAGAAGAACGATATATATTGATTCAATTGATGATGTAGAAGAACAGGTTGTACCACAACATAGCCAAATGTTGGGTGGAACAACAGAATACACTGATTCAGATGCTGACATAAAAGAACAAGTAAAGCAAGCTATATTCGAAACAGATAGGAAGAAAACAAATGGAACATATCTTTGGTTGGAAAAAATTGTAATTGGTTTTAATATGGGAATCTCTTCCCGTTTTCAAGTTTTACTGAAACAAACAGTTTGCCCTATTAAAGTGAAACGGTATAACTCTTATAAGAAAGTCTACGAAAACTGTAAGGGTTATGGAGATTTGAAAAACTGTACCGTTGAATACAAATATTTCGATCCAACCATATCAACTGTTGAATGTTAAACCACAAAGCAAAGATAACATTACACCAATTACTTTTCATCTTTAAATAAAGAAACCATTTTGGAATTTTGATTTATAAAGAAAGTAGAAGAATCGATAATT | IEMLGGTTLHGRDAAVMLPEAQEALFLSDTKQKFGVFHKLVRVEEVSTMGIFTIMQVVVQNTDCQVSHDIFSYNDVLKKCRAESEKQKCRIEYKYHDPLTATALCVKKVEESIIIPQRDQRANRRRTIYIDSIDDVEEQVVPQHSQMLGGTTEYTDSDADIKEQVKQAIFETDRKKTNGTYLWLEKIVIGFNMGISSRFQVLLKQTVCPIKVKRYNSYKKVYENCKGYGDLKNCTVEYKYFDPTISTVEC | many | many | TSC0210  TSC1941 |
|  |  |  |  |  |  |
| *sml-3*/  5_132_2 | atgtcagttttagcagcttttatcttctttttcatggcagttatgcctgaaatcaatgcggatttgagtccattggaagaagcccaaggttacatataccaatctgatttacaaagcggtaaaggtcatttccgcagagttctcgatgtaagcgatgtcgacacaagtgacggattatccttaacgatagacgctcttccaactacatgtcctgtgtcatcagaaatgactcaagaacaagtgtattcagatgagtgccccgtcactaaagacgaatacgaccaaatagaatgccgtttggagcttaaccattctaaaactggccaaattgaatgtacatattatgggcat | MSVLAAFIFFFMAVMPEINADLSPLEEAQGYIYQSDLQSGKGHFRRVLDVSDVDTSDGLSLTIDALPTTCPVSSEMTQEQVYSDECPVTKDEYDQIECRLELNHSKTGQIECTYYGH | many | many | TSC00935  TSC00437 |
| 5_132_1 | atgtcagttttagcagcttttatcttctttttcatggcagttatgcctgaaatcaatgcggatttgagtccattggaagaagcccaaagttacatataccaatctgatttgcaaagcggtaaaggtcatttccgcagagttctcgatataagcgatgtcgacacaagtgacggattatccttaacgatagacgctcttccaactacatgtcctgtgtcatcagaaatgactcaagatcaagtgtattcagatgagtgccccgtcaccagagaggaatatgacgaaatagaatgccatttgaagcttgaccattctaaaactggccaaattgaatgtacatattatggacat | MSVLAAFIFFFMAVMPEINADLSPLEEAQSYIYQSDLQSGKGHFRRVLDISDVDTSDGLSLTIDALPTTCPVSSEMTQDQVYSDECPVTREEYDEIECHLKLDHSKTGQIECTYYGH | ps93f10.y1 | **BQ542128** | TSC00935 |
| 5_149 | atgtccgcttcgaatctagttattatttcatttttgatatgcactattgcgattattaatattcatggagagttggatattttgcaatatgcaaaacaaacaatttatgcatcagatttgaaaaaacccgatcaaaattttcgaaaagttgtttctgtggatagttcaaccgctgctggcgaagacattgtcagtataacaattcatgccaaagaaacaacttgtgcagtgtcacaacaagtttgcaaaatagattataaagtgtattcaaaagaatgcacagtttctacgaggaaatacgaagatattgaatgcttcttgacagcaaatagtgacagagttggcactctgaaatgtatttatcaaacaaaacattgaa | MSASNLVIISFLICTIAIINIHGELDILQYAKQTIYASDLKKPDQNFRKVVSVDSSTAAGEDIVSITIHAKETTCAVSQQVCKIDYKVYSKECTVSTRKYEDIECFLTANSDRVGTLKCIYQTKH | ps92d06.y1 | **BQ542046** | TSC03373 |
| 7_83 | atgtcagttttagcagcttttatcttctttttcatggctgttatgcctgaaatcaatgcagattcgagtgcattggaagaagccaaagattacatttaccaatctgatttgcaaagcggtaaaggttatttccgcaaagttcgcagtgtaagcgaagtcgacagaagtgaaggattatccttaacaatagatgctctttcaactacatgccaagtgtcatcagaaattagtcaagaacaggtgtattctgatgagtgccccatcacgaaagtggaatacgacgaaatagaatgccatttgaagcttgaccattctaaaactggccagctggaatgcacatattatggagcataaa | MSVLAAFIFFFMAVMPEINADSSALEEAKDYIYQSDLQSGKGYFRKVRSVSEVDRSEGLSLTIDALSTTCQVSSEISQEQVYSDECPITKVEYDEIECHLKLDHSKTGQLECTYYGA | ps79b06.y1 | **BQ542660** | TSC02648 |
| 5_151 | atgtcagttttagcagcttttatcttctttttcatggcagttatgcctaaaatcaatgcggatttgagtccattggaagaagcccaaggttacatataccaatctgatttgcaaagcggtaaaggtcatttccgcagagttctcgatgtaagcgatgtcgacacaagtgacggattatccttaacgatagacgctcttccaactacatgtcctgtgtcatcagagatgactcaagaacaagtgtattcagatgagtgccccgtcactaaagacgaatacgaccaaatagaatgccgtttggagcttaaccattctaaaactggccaaattgaatgtacatattatggatat | MSVLAAFIFFFMAVMPKINADLSPLEEAQGYIYQSDLQSGKGHFRRVLDVSDVDTSDGLSLTIDALPTTCPVSSEMTQEQVYSDECPVTKDEYDQIECRLELNHSKTGQIECTYYGY | ps12b01.y1  ps99c10.y1 | **BG321994**  **BQ576749** | TSC00437 |
| 5_88_1 | atgtcaattttatcagttctagtcttttttctcatggcattcatgcctgaagtcagtgcagattcggatgcaatgaaagaagcccgaagttacatttactcatctgatttgcaaaaaccggatcaaaattttcgcaaagttacctcagtaagcaaagtcgacagaagcgatggtttgtccatggagatggatgtagcccaaacttcatgtcaagtttcatcaaaacttagtcaagaaaaagtttatacagatgcttgccccgtatctaacgaatatgaaaaaataaaatgcaatctgaagcttgatgagaacaaaaatggccagttaaaatgtgtatacgttgaagcg | MSILSVLVFFLMAFMPEVSADSDAMKEARSYIYSSDLQKPDQNFRKVTSVSKVDRSDGLSMEMDVAQTSCQVSSKLSQEKVYTDACPVSNEYEKIKCNLKLDENKNGQLKCVYVEA |  |  |  |
| 5_88_2 | atgtcagctttagcagcttttatcttctttttcatggcagttatgcctgaaatcaatgcggatttgagtgaattggatgaagctaaaaattacatttaccaatctgatttacaaaccggtagaggtaatttccgcaaagttctcaaagttagaaatgtcgacacaagtgacggattatccttaacgatagacgctcttccaactacgtgccctgtgtcatcagaaaagagtctagaagaggtgtattcagatgagtgccgcatcaccaaagatgaatacgacaaaatagaatgccatttaaaacttgaccagaataaatctggccaaattgaatgcacatattatgcagtc | MSALAAFIFFFMAVMPEINADLSELDEAKNYIYQSDLQTGRGNFRKVLKVRNVDTSDGLSLTIDALPTTCPVSSEKSLEEVYSDECRITKDEYDKIECHLKLDQNKSGQIECTYYAV | MBTsMLW126T7SEQ | **BG354772** | TSC00437 |
| 0_23 | atgtcattgaaatatttttgtctctgtcttctactatgtttcattggtgttggtagtgataattacagcgaaggttttcttgattttgcatcaagaattgtctatcaggcagatacattgcagtctaatagccattatcgaaatgttatccgtgcacgtagagaagactcagaagatggactgagattgaaattacttgtcatcgaaacagtatgctccacagaaagagcattgtttataggatatgtttattcttataactgccctaattccagtgcattctctagtattaaatgcttgctaacaatgaataataatcgaacgaatattgtattgagatgtaattatcttcgtgttgaacgtatttaa | MSLKYFCLCLLLCFIGVGSDNYSEGFLDFASRIVYQADTLQSNSHYRNVIRARREDSEDGLRLKLLVIETVCSTERALFIGYVYSYNCPNSSAFSSIKCLLTMNNNRTNIVLRCNYLRVERI | ps56h08.y1 | **BG521196** | TSC02335 |

The *sml-2* and *3* family members identified in the *T. spiralis* genome assembly are shown with their cDNA sequence and the predicted protein translation. Expressed sequence tag (EST) clones, their GenBank accession numbers and clusters representative of these genes are also shown.
